# Farm Houses

**Published:** 1866-04

**Authors:** 


### FARM HOUSES.

There can be no good reason why a piazza from ten to fourteen feet broad should not extend the whole front, end, and part of the rear of every farm-house; and considering the personal advantages of such an arrangement, and the air of coolness and beauty and liveliness which they present in summer, it must be put down as a great oversight, in that they are not more common than they are. It can not be denied that they contribute greatly to the coolness of the lower rooms in warm weather, and afford facilities for play to the children in inclement or muddy weather, and for exercise to grown persons, which are of inestimable value in promoting health. It would surprise most persons greatly to know how many girls in the country have fixed diseases grafted on them before they leave their teens; this is more strikingly the case with the daughters of farmers who are "well off" and actually rich. This comes about largely from the fact that they have not the facilities of exercise half equal to similar classes in large towns and cities. They, perhaps, sweep a room, or dust the parlors, or make up a bed or two in the morning; and that is about all the exercise they take on foot during the day, except when they have visitors; the remainder of the time they sit and sew, or read, or loll about, not altogether because they do not want to exercise themselves, but because there are not the facilities of doing so. Few farmers have a spare horse suitable for a girl to ride, and if they did, she must have some one to ride with her; that requires a second horse, and the brother or father must accompany her. These circumstances narrow down the chances of horseback exercise, exclusive of church-going days, to about a dozen or two hours in a year to eleven farmers' daughters in a dozen. And however inclined to walk, it is impracticable in winter, because they must step from the door-sill into mud, or slush, or snow. In summer it is too hot in the middle of the day; in the morning the grass is bedewed; and so in the evening, unless it is early, say just before sundown, when it is not altogether safe to be out of sight of the house. If there were commodious piazzas, there would be admirable facilities for walking at all seasons, and every day, for games, rope-jumping, plays, and promenades of every description; and by reducing it to a system, an amount of exercise in the open air could be taken every day, the value of which upon the physical health, the mental power, and general vivacity, can not be readily estimated.

